# The chaperone DnaK controls the fractioning of functional protein between soluble and insoluble cell fractions in inclusion body-forming cells

**DOI:** 10.1186/1475-2859-5-26

**Published:** 2006-08-07

**Authors:** Nuria González-Montalbán, Elena García-Fruitós, Salvador Ventura, Anna Arís, Antonio Villaverde

**Affiliations:** 1 Institut de Biotecnologia i de Biomedicina, Universitat Autònoma de Barcelona, Bellaterra, 08193 Barcelona, Spain; 2 Departament de Genètica i de Microbiologia, Universitat Autònoma de Barcelona, Bellaterra, 08193 Barcelona, Spain; 3 Departament de Bioloquímica i de Biologia Molecular, Universitat Autònoma de Barcelona, Bellaterra, 08193 Barcelona, Spain

## Abstract

**Background:**

The molecular mechanics of inclusion body formation is still far from being completely understood, specially regarding the occurrence of properly folded, protein species that exhibit natural biological activities. We have here comparatively explored thermally promoted, *in vivo *protein aggregation and the formation of bacterial inclusion bodies, from both structural and functional sides. Also, the status of the soluble and insoluble protein versions in both aggregation systems have been examined as well as the role of the main molecular chaperones GroEL and DnaK in the conformational quality of the target polypeptide.

**Results:**

While thermal denaturation results in the formation of heterogeneous aggregates that are rather stable in composition, protein deposition as inclusion bodies renders homogenous but strongly evolving structures, which are progressively enriched in the main protein species while gaining native-like structure. Although both type of aggregates display common features, inclusion body formation but not thermal-induced aggregation involves deposition of functional polypeptides that confer biological activity to such particles, at expenses of the average conformational quality of the protein population remaining in the soluble cell fraction. In absence of DnaK, however, the activity and conformational nativeness of inclusion body proteins are dramatically impaired while the soluble protein version gains specific activity.

**Conclusion:**

The chaperone DnaK controls the fractioning of active protein between soluble and insoluble cell fractions in inclusion body-forming cells but not during thermally-driven protein aggregation. This cell protein, probably through diverse activities, is responsible for the occurrence and enrichment in inclusion bodies of native-like, functional polypeptides, that are much less represented in other kind of protein aggregates.

## Background

In bacteria, formation of inclusion bodies is common during overexpression of plasmid-encoded recombinant genes, and this fact represents an important matter of concern in biotechnology [1]. Like in mammalian aggresomes, inclusion body formation is stimulated when proteolysis is impaired in protease-deficient mutants [2,3], and these protein deposits act as reservoirs of misfolded polypeptide chains [4] for their further refolding or proteolysis [3,5,6]. Bacterial inclusion bodies are dynamic structures, they grow resulting from an unbalanced equilibrium between constant protein deposition and removal that is lost in absence of protein synthesis [4,7]. Intriguingly, they contain significant amounts of protein in a native-like form [8-12], a fact that is reflected by the important extent of biological activity exhibited by inclusion bodies formed by very different target proteins [13-15]. Why active protein is found in inclusion bodies is still controversial, and the mechanics of the aggregation process that involves properly folded polypeptides (or polypeptides with properly folded domains critical for activity) remains obscure. In this context, it has been recently proposed that protein aggregation in bacteria is not an all-or-nothing process [16], since the quality of recombinant proteins extends over a continuum of conformational forms [17], that include soluble aggregates [18,19] and active protein entrapped in true, refractile inclusion bodies [9,13]. The conformational status of the inclusion body protein is influenced, among others, by environmental factors such as the growth temperature [20] and the gene expression strategy [21], but little is known about the role of cellular factors on the quality of protein species in both soluble and insoluble cell fractions.

In this work, we have explored the occurrence of active, properly folded polypeptides in inclusion bodies and in thermally driven aggregates formed by the same protein species, and the influence of the main chaperones DnaK and GroEL in the quality of the deposited polypeptides but also of those remaining in the soluble fraction. Intriguingly, while both type of aggregates display a few common physiological traits, the occurrence of active protein species is much higher in inclusion bodies, at expenses of a poorer quality (when compared to thermal aggregates) of the protein population remaining in the soluble fraction. Also, the chaperone DnaK has a main role in the distribution of active polypeptides between soluble and insoluble cell fractions in inclusion body forming cells but not during thermally driven protein aggregation.

## Results

### Composition of β-galactosidase-based thermal aggregates and inclusion bodies

**Figure 1:**
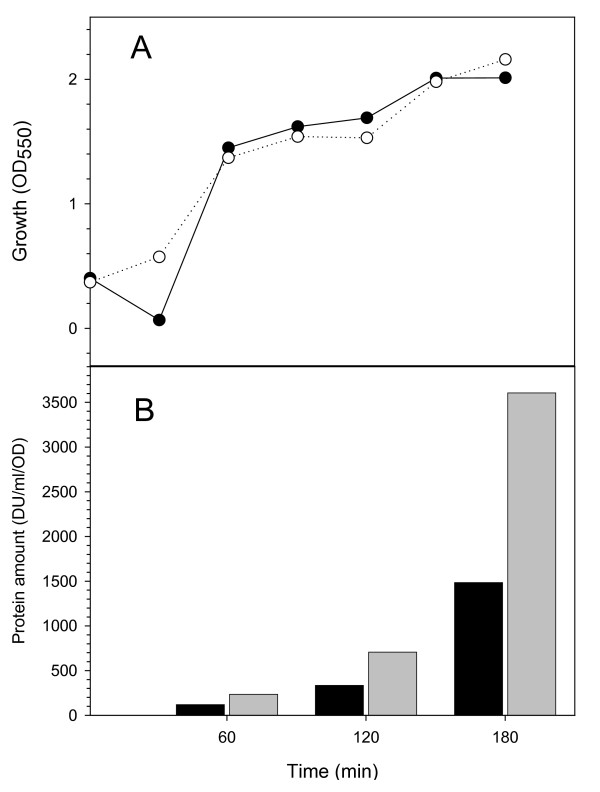
A. Cell growth measured through optical density for MC4100 cultures producing either β-galactosidase (black symbols) or VP1LAC (white symbols). Time 0 represents the temperature up shift. B. Total yield of β-galactosidase (black bars) and VP1LAC (grey bars), as measured by Western blot densitometric units.

**Figure 2:**
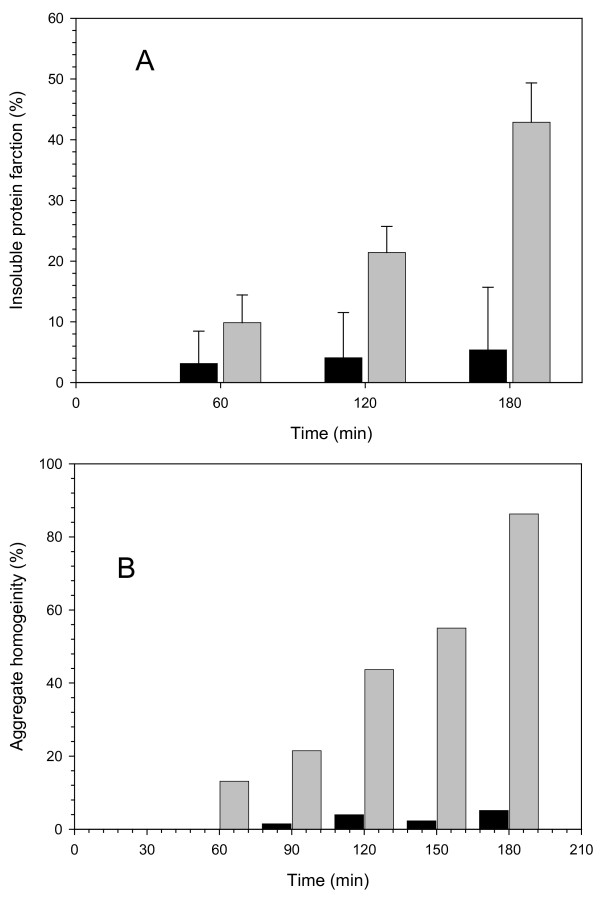
A. Fraction of the produced recombinant polypedptides found in protein deposits, either thermal aggregates of β-galactosidase (black bars) or VP1LAC inclusion bodies (grey bars). B. Percentage of β-galactosidase (black bars) and VP1LAC (grey bars) found in thermal aggregates and inclusion bodies respectively.

**Figure 3:**
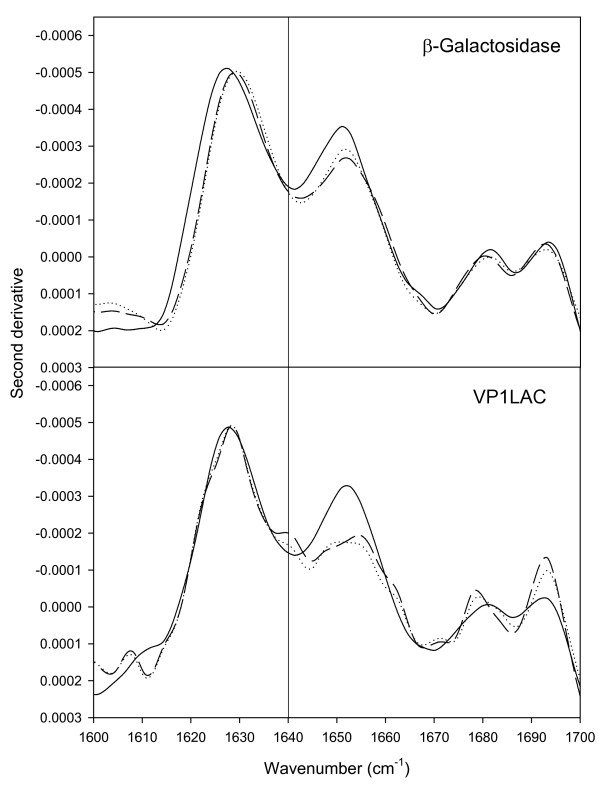
FTIR of β-galactosidase aggregates (top) and VP1LAC inclusion bodies (bottom) formed during either 1 (continuous), 3 (dotted) or 5 (dashed) hours. The vertical line at 1640 cm^-1 ^indicates the position of the band that can be attributed to intramolecular β-sheet.

*E. coli *β-galactosidase is a huge, homotetrameric enzyme formed by the *lacZ *gene product. When overproduced in bacteria, the enzyme remains soluble in the cell cytoplasm and is clearly functional. In an engineered version of the enzyme, the VP1LAC fusion, the presence of a small viral capsid protein at the amino terminus promotes aggregation as cytoplasmic inclusion bodies, and VP1LAC is distributed in the soluble and insoluble cell fractions at comparable proportions [22]. Interestingly, VP1LAC inclusion bodies are enzymatically active [13] at an extend not much different than that found in the soluble protein version [21]. To compare the performance of the enzyme in either thermal aggregates and inclusion bodies, we have used a particular thermo-inducible expression system that enables a comparative study. Expression of both *lacZ *and *VP1LAC *genes was triggered from a temperature-inducible plasmid vector encoding a temperature sensitive lambda repressor, essentially inactive at 42° [23]. The temperature shift from 28 to 42° induced efficient recombinant protein production (without signs of cell toxicity) (Figure 1A), The lower amounts of β-galactosidase compared to that of VP1LAC (Figure 1B) were probably caused by a slightly higher proteolytic sensitivity of the parental protein as previously reported [24]. Under this conditions, cells undergo a mild heat shock that results in thermal denaturation and aggregation of cellular proteins. In particular, the production of the misfolding prone VP1LAC resulted in its accumulation as inclusion bodies [4]. Also, a small part of the recombinant β-galactosidase present in the cells (up to around 5%) was found in the insoluble cell fraction as part of thermal aggregates, and this figure remained nearly constant throughout the heat shock (Figure 2A). In contrast, a progressively higher fraction of VP1LAC (up to 45% at 3 h) occurred as inclusion bodies (Figure 2A). Despite at 42°C the recombinant β-galactosidase is the most abundantly produced protein in the cell, the enzyme only represented around 3% of the protein species found in the insoluble cell fraction, while VP1LAC accounted for 90% of the inclusion body material (Figure 2B). During the experiment time, inclusion bodies were steadily enriched with VP1LAC species and therefore their homogeneity dramatically increased, while the β-galactosidase fraction in thermal aggregates randomly fluctuated between 1.5 and 3%. These results are compatible with the seeding process recently shown to drive inclusion body formation [9] and indicate that, in contrast, thermal aggregation does not involve interaction between homologous protein patches and it is not, at least strictly, sequence-specific. On the other hand, polypeptides embedded in both kinds of aggregates undergo important changes in their global secondary structure (Figure 3; Table 1), through the continuous formation of extended, intermolecular β-sheet structure, being more pronounced in inclusion bodies than in thermal aggregates. This was deduced from the evolution of the bands at 1627 cm^-1 ^and 1692 cm^-1 ^(β-sheet) relative to that at 1652 cm^-1 ^(disordered and/or α-helix) (Table 1). The presence of a band at 1638–1640 cm^-1^, even if not well resolved, can be attributed to the occurrence of some intramolecular β-sheet. This band appeared only in aged inclusion bodies and it was absent in thermal aggregates. According to previous analysis [9] this peak corresponds to native-like species, that could be accounted by β-galactosidase moieties.

**Table 1:** Time evolution of the secondary structure in both β-galactosidase thermal aggregates and VP1LAC inclusion bodies as measured by FTIR peak ratios.

Protein^a^	Time (h)	Ratio 1627/1652^b^	Ratio 1692/1652^b^
β-galactosidase	1	1.31	0.42
	3	1.53	0.40
	5	1.63	0.52

VP1LAC	1	1.36	0.54
	3	2.14	0.83
	5	2.21	0.96

^a^Data are from Figure 3

^b^Peaks at 1627 cm^-1 ^and 1692 cm^-1 ^can be attributed to extended intermolecular β-sheet while that at 1652 to disordered structure and/or α-helix.

### Impact of DnaK and GroEL in β-galactosidase aggregation and activity

**Figure 4:**
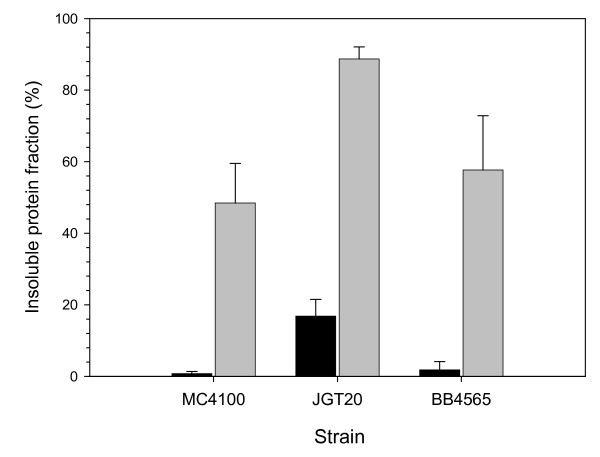
Percentage of the recombinant protein found in protein deposits, either thermal aggregates of β-galactosidase (black bars) or VP1LAC inclusion bodies (grey bars), in MC4100 (wild type), JGT20 (DnaK^-^) and BB4565 (GroEL44) strains.

**Figure 5:**
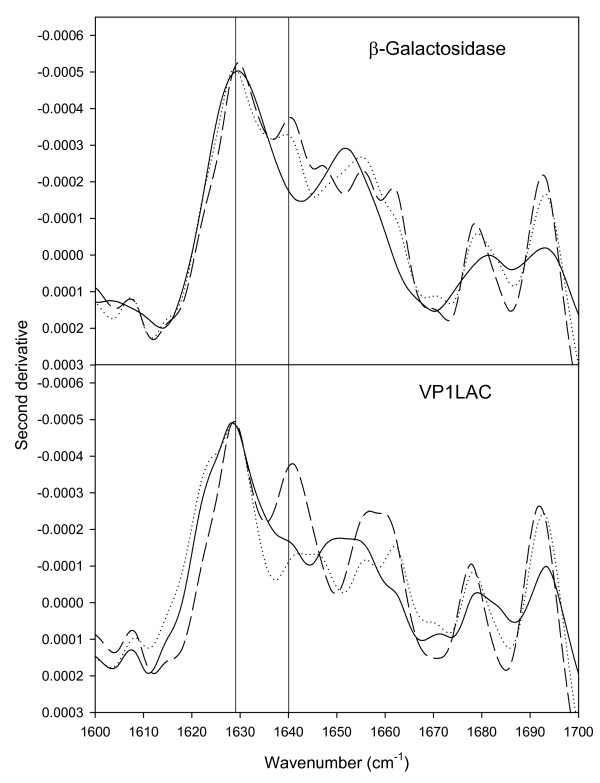
FTIR of β-galactosidase aggregates (top) and VP1LAC inclusion bodies (bottom) formed in MC4100 (continuous), JGT20 (dotted) and BB4565 (dashed) strains. Vertical lines at 1628 and 1640 cm^-1 ^indicate intermolecular β-sheet and intramolecular β-sheet, respectively.

The formation of β-galactosidase thermal aggregates and VP1LAC inclusion bodies was explored in absence of the main cytoplasmic chaperones, either DnaK or GroEL. It has been previously reported that when DnaK is not available, inclusion bodies are larger than in the wild type strain and the amounts of soluble VP1LAC much lower [25]. Such alteration in inclusion body formation can be accounted for by two described DnaK activities, namely preventing aggregation [26] or actively disaggregating proteins [27-29], both done in combination with other chaperones and small heat shock proteins. As observed in the DnaK^- ^background (Figure 4), the deposition of the recombinant enzyme was enhanced in both types of aggregates, although the negative impact on solubility was dramatically higher in those formed by the parental form of the enzyme. The parallel stimulation of aggregation would indicate that DnaK is managing both thermal aggregates and inclusion bodies, although the chaperone could be more active in controlling deposits of denatured polypeptides. This is suggested by the fact that the amount of insoluble VP1LAC is not even doubled in its absence, while the increase of aggregated β-galactosidase is nine fold higher than that of the wild type enzyme. The presence of a non-functional form of the chaperone GroEL (GroEL44), only had a minor, non-significant impact on protein solubility in both aggregation conditions (Figure 4). The comparative ATR-FTIR of both types of aggregates formed in the mutant strains indicated a different structural pattern compared to the wild type (Figure 5). For VP1LAC in inclusion bodies, the absence of GroEL results in a significant enrichment of native like intramolecular β-sheet structures (peaking at 1638–1640 cm^-1^). In the case of thermal aggregates the absence of either DnaK or GroEL results in more complex FTIR spectra relative to that recorded for the aggregates formed in the wild-type strain, reflecting a higher degree of conformational heterogeneity.

As it has recently been proven that deposition as bacterial inclusion bodies does not necessarily represent functional protein inactivation [13], the specific activity of both model proteins was investigated in wild type cells and in absence of either DnaK or functional GroEL. As expected (Table 2), the soluble β-galactosidase was more active (from 2 to 8 fold) than the soluble VP1LAC. Despite this fact, protein aggregated as inclusion bodies was much more active than that occurring in thermal aggregates (up to 10 fold in wild type cells), indicating a higher occurrence of properly folded protein. While GroEL seems to be poorly relevant, this fact is clearly depending on DnaK, since in JGT20, insoluble VP1LAC is around 10 fold less active than insoluble β-galactosidase.

**Table 2:** Specific activity (in U/ng) of β-galactosidase and its derivative VP1LAC produced in different strains, in the soluble and insoluble cell fractions.

Strain	Soluble fraction	Insoluble fraction
MC4100/pJCO46	628.2 ± 40.5	6.3 ± 0.3
MC4100/pJVP1LAC	234.1 ± 52.9	65.2 ± 19.4
BB4565/pJCO46	689.7 ± 164.9	63.6 ± 2.2
BB4565/pJVP1LAC	230.2 ± 25.7	129.6 ± 45.9
JGT20/pJCO46	888.9 ± 179.3	175.2 ± 34.9
JGT20/pJVP1LAC	12.5 ± 3.8	10.3 ± 6.3

### Physiological disintegration of thermal aggregates and inclusion bodies

**Figure 6:**
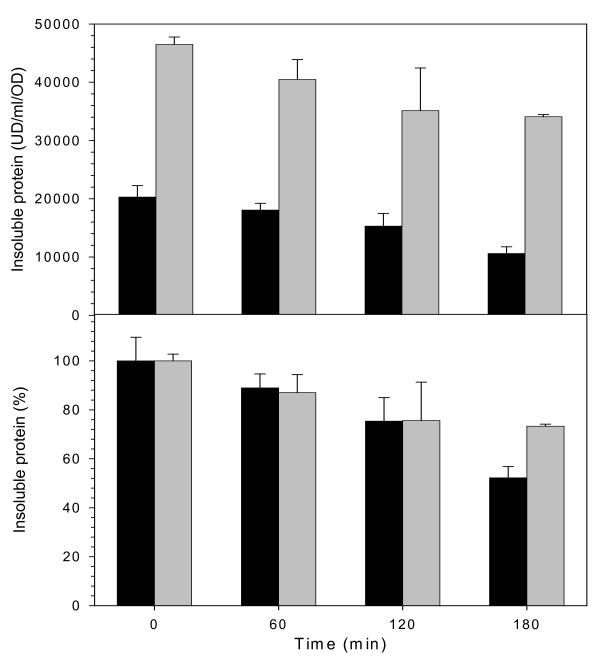
A. Amount of β-galactosidase (black bars) or VP1LAC (grey bars) retained in the insoluble cell fraction after arrest of protein synthesis, as determined by Western blot densitometric units. B. Representation of the above values referred to the starting insoluble material amount.

The kinetics of physiological disintegration of inclusion bodies and thermal aggregates were compared upon arrest of protein synthesis to investigate the cell ability to process both kinds of structures when chaperones and proteases become available. As shown in Figure 6, the protein removal process is similarly efficient on both aggregate types, although inclusion body disintegration might be slightly delayed from 3 hours on, with respect to the disintegration of denatured protein clusters.

## Discussion

Under mild heat-shock conditions, most of a recombinant β-galactosidase produced in *E. coli *remains in the soluble cell fraction, while an engineered derivative containing an aggregation-prone viral peptide (VP1LAC), forms cytoplasmic inclusion bodies. Up to around 45% of the produced VP1LAC is found trapped in such structures (Figure 2). When comparing with thermal aggregation, the formation of bacterial inclusion bodies appears as a highly specific event, that renders homogenous particles species regarding composition (90% purity in inclusion bodies versus 5% in thermal aggregates, Figure 2). The heterogeneous nature of *in vivo *formed thermal aggregates was not unexpected as many termolabile cellular proteins are deposited as misfolded versions at high temperatures [30]. The high purity of inclusion bodies, however, is reached only 3 hours after inducing gene expression and before that, these particles are progressively gaining homogeneity (Figure 2). In agreement to previous observations [4,5,7], this fact reflects the dynamic nature of inclusion bodies versus the poor evolution of β-galactosidase present in thermal aggregates, despite this protein is much more abundant in the cell than any of the other deposited species. The seeding mechanics of inclusion body formation [9] and the sequence-dependent aggregation determinants acting there [9,31] have not been described in thermal aggregation, and their absence could account for the different time-dependent composition patterns.

However, ATR-FTIR analysis shows that polypeptides embedded in both kinds of aggregates undergo a structural evolution during formation (Figure 3, Table 1) that can be seen as a continuous formation of new, non-native, extended intermolecular β-sheet structure, more pronounced in inclusion bodies than in thermal aggregates.

The presence of native-like intramolecular β-sheet structure in inclusion bodies aged 3 and 5 h, and absent in the thermal aggregates (peaking at 1638–1640, Figure 3), would be indicative of the presence of a fraction of properly folded proteins or protein domains, in agreement with previous structural analysis [8,10-12,32]. Also, although aggregation reduces the β-galactosidase activity in both β-galactosidase and VP1LAC (Table 2), many descriptions of biological activity in structurally different inclusion body proteins [13-15,21,33,34] indicate that the presence of active protein could be a general trait of such protein deposits. In fact, we prove here that inclusion body protein is 10-fold more active than its thermally denaturated counterpart (Table 2). On the other hand, the disintegration of inclusion bodies and thermal aggregates upon arrest of protein synthesis shows comparable rates (Figure 6). This fact indicates that both aggregate types are under the surveillance of disaggregating chaperones [27-29,35,36] and/or proteases [3,4]. Protein removal in both kind of aggregates also suggests that physiological disaggregation is not specifically involving residual native-like structure, as it occurs also on heat denaturated protein in which the presence of properly folded polypeptide backbones cannot be detected (Figure 3). Contrarily, the possibility of refolding (or digestion) specifically targeted towards misfolded polypeptides needs to be explored.

Interestingly, the lack of either GroEL or DnaK major cytosolic chaperones globally enhances the activity of the aggregated proteins in both thermal deposits and inclusion bodies (Table 2). The comparative FTIR analysis of both type of aggregates formed in the mutant strains indicates a different general structural pattern compared to the wild type (Figure 5). Aggregates formed in the absence of chaperones are more heterogeneous than those in the wild type strain. The presence of native-like intramolecular β-sheet structure (peaking at 1638–1640 cm^-1^), corresponding to native-like VP1LAC in inclusion bodies is enriched specially in the absence of functional GroEL. This coincides with an increased activity of this aggregates, suggesting that this signal corresponds to the accumulation of native and functional β-galactosidase [9]. For thermal aggregates, the presence of a band in the region assignable to intramolecular β-sheet conformations is also detected in the absence of both chaperones. Although, due to the heterogeneous composition of this aggregates, the band cannot attributed to a unique protein species, the significant increased enzymatic activity exhibited by thermal aggregates produced in the absence of chaperones suggests that native functional β-galactosidase contributes, at least partially, to this band in the FTIR spectra.

On the other hand, the specific activity of soluble VP1LAC is between 2 and 3 fold lower than that of the parental enzyme (for wild type and GroEL44 strains), as it would be expected for a fusion protein. However, in absence of DnaK, soluble VP1LAC (but not β-galactosidase) is much more inactive, indicating that this chaperone importantly participates in the VP1LAC (but not β-galactosidase) folding process as previously suggested [21]. Also, the specific activity of inclusion body VP1LAC is surprisingly higher than that of denaturated β-galactosidase, only when DnaK is present (Table 2). This intriguing observation indicates an enrichment of inclusion body active species in which DnaK might have a positive role. It cannot be discarded that DnaK, acting as a disaggregase at inclusion body's surface [37], could selectively remove inactive (misfolded) protein. Alternatively, DnaK could preferentially prevent the incorporation of inactive protein into inclusion bodies. In the case of β-galactosidase, the presence of DnaK modulates the deposition of the enzyme under heat stress, as shown by the nine fold increase of β-galactosidase in the aggregated fraction in the absence of this chaperone. The low activity and amount of β-galactosidase in thermal aggregates suggest that they are formed by highly aggregation-prone protein conformations which escape DnaK control. In a DnaK^- ^background, this control does not longer exist and a more heterogeneous set of polypeptide conformations, including some functional or partially functional ones, can aggregate as thermal deposits. This is in accordance both with the higher conformational heterogeneity, as seen by FTIR, and the higher activity of thermal aggregates in the absence of DnaK.

Altogether, these observations point out significant differences between inclusion body formation and *in vivo *thermal aggregation, as revealed by a convenient comparative expression system. While both types of aggregates are controlled by the quality cell system, inclusion bodies are homogeneous and highly organized structures progressively enriched in properly folded versions of the main protein component.

## Conclusion

The formation of both protein deposits induced in bacteria by heat shock and inclusion bodies is negatively controlled by DnaK, and both type of aggregates efficiently disintegrate when the conformational stress is over. Despite such similarities, inclusion bodies are more homogeneous in composition and result progressively enriched in native-like forms of the target protein during their construction, what results in a detectable evolution of the global secondary structure of the embedded proteins. In this regard, precipitation as inclusion bodies keeps the target protein in a more functional form than in thermal aggregates, but only when DnaK is present. Interestingly, the biological activity of the soluble counterparts is especially poor when inclusion bodies are more active, suggesting that active polypeptides from the soluble cell fraction are used for inclusion body construction. Therefore, this particular chaperone is important to ensure the biological activities of inclusion body polypeptides that are not conserved in other aggregation conditions, by controlling the distribution of functional protein species between soluble and insoluble cell fractions. Protein packaging as bacterial inclusion bodies is then a cell driven deposition process.

## Methods

### Bacterial strains, plasmids, proteins and gene expression conditions

Recombinant proteins were produced in *Escherichia coli *MC4100 *araD139 Δ(argF-lac*) *U169 rpsL150 relA1 flbB5301 deoC1 ptsF25 rbsR*, and their derivatives GroEL44 *groEL44 zdj::Tn10 zje::kan *(BB4565) and DnaK^- ^*dnaK thr::Tn10 *(JTG20). Plasmid pJCO46 encodes a soluble, pseudo-wild type *E. coli *β-galactosidase, and the closely related pJVP1LAC, a derivative β-galactosidase fusion protein containing the aggregation-prone VP1 capsid protein of foot-and-mouth disease virus joined at the amino terminus [22]. The presence of the viral protein segment promotes aggregation of the whole fusion and under our gene expression conditions, approximately 50% of VP1LAC is found as cytoplasmic inclusion bodies. Both *lacZ *and *VP1LAC *genes are under the control of tandem lambda *p*_*L*_*p*_*R *_lytic promoters and repressed by a plasmid-encoded and constitutively expressed temperature-sensitive CI^857 ^repressor. Bacterial cells were cultured in shake flasks up an OD_550 _of 0.3, in Luria-Bertani (LB) rich medium [38] with 100 μg/ml ampicillin. Then, the expression of both *lacZ *and *VP1LAC *genes was triggered by temperature up-shift from 28 to 42°C. When required, protein synthesis was arrested by adding chloramphenicol at 200 μg/mL and the cultures were further incubated at 28°C. Usually, data were obtained from three or more independent experiments.

### Quantitative protein analysis

Samples of bacterial cultures (10 ml) were low-speed centrifuged (15 min at 12000 g) and cell pellets resuspended in denaturing buffer. For the analysis of soluble and insoluble cell fractions, samples were resuspended in 500 μl of Z buffer without β-mercaptoethanol [39] with one tablet of protease inhibitor cocktail (Roche, ref. 1 836 170) per 10 ml buffer. Such mixtures, once jacketed in ice, were sonicated for a minimum of 5 min at 50 W under 0.5 s cycles, and centrifuged for 15 min at 12000 g. Soluble and insoluble fractions were separately resuspended in denaturing buffer [40] for Western Blot and Coomassie blue staining. After boiling for 20 min, small sample volumes were loaded onto gels. For Western blot, a rabbit anti β-galactosidase sera was used to immunodetect both β-galactosidase and VP1LAC proteins. Full-length forms of VP1LAC and its major proteolysis fragments (both know to be functional) were considered in the analysis. Dried gels and blots were scanned at high resolution and bands quantified by using the Quantity One software of Bio Rad. All determinations were done at least in quadruplicate.

### Conformational analysis by ATR-FTIR spectroscopy

For ATR-FTIR spectroscopy analysis, inclusion bodies and thermal aggregates were purified from cell extracts by repeated detergent washing as described [41]. Then, both kinds of aggregates were dried for two hours in a Seed-Vac system before analysis to reduce water interference in the infrared spectra. A Bruker Tensor 27 FT-IR Spectrometer (Broker Optics Inc.) with a Golden Gate MKII ATR accessory (Specac) was employed for ATR FT-IR experiments. Each spectrum comprises 16 scans measured at a spectral resolution of 4 cm^-1 ^in the 4000–600 cm^-1 ^range. Spectral data were acquired with OPUS MIR Tensor 27 software version 4.0 (Broker Optics Inc.). All the absorbance spectra were normalized to correct for concentration dependent effects and the second derivatives of the amide I band spectra were used to determine the frequencies at which the different spectral components were located.

### Determination of the specific activity

To determine the specific activity of both soluble and aggregated β-galactosidase and VP1LAC proteins, 2.5 ml culture samples were disrupted by sonication as described [42] and centrifuged for 15 min at 15000 g. The soluble fraction was directly used for the analysis, and inclusion bodies and thermal aggregates were purified from cell extracts by repeated detergent washing [41]. Substrate hydrolysis was quantified espectrophotometrically as described [21] and the amounts of recombinant protein either soluble or within the aggregates, was specifically determined by Western blot as indicated above, by using serial dilutions of a commercial β-galactosidase of known concentration as pattern. All determinations were done in triplicate.

## Abbreviations

ATR Attenuated total reflection

FTIR Fourier transformed infrared

LB Luria-Bertani

## Competing interests

The author(s) declare that they have no competing interests.

## Authors' contributions

NGM performed most of the experimental and Figures, EGF analysed the biological (and specific) activities of recombinant proteins, SV and AA designed the experimental and analysed structural data and AV directed the work and prepared the manuscript.
